# In vitro evaluation of the efficacy of a new orthodontic primer in metallic brackets cementation

**DOI:** 10.1007/s00784-026-06896-7

**Published:** 2026-05-07

**Authors:** Byron Carpio–Salvatierra, Mayra Alejandra Nuñez, Pâmela Maria Kusdra, Victor Martins Montalli, Ana del Carmen Armas Vega, Paulo Vitor Farago, Alessandro D. Loguercio

**Affiliations:** 1 Department of Restorative Dentistry, School of Dentistry, State University of Ponta Grossa, Ponta Grossa, Paraná Brazil; 2 Department of Dentistry and Dental Materials, Universidad San Francisco de Quito, Quito, Pichincha, Ecuador; 3 Department of Orthodontics, School of Dentistry, Faculdade São Leopoldo Mandic, Curitiba, Paraná Brazil; 4 Department of Orthodontics, School of Dentistry, Faculdade São Leopoldo Mandic, Campinas, Sao Paulo, Brazil; 5 Facultad de Ciencias de la Salud, Departamento de Odontología, Universidad de los Hemisferios, Quito, Pichincha Ecuador; 6 Department of Pharmaceutical Science, School of Pharmacy, State University of Ponta Grossa, Ponta Grossa, Paraná Brazil; 7 Rua Carlos Cavalcanti 4748 Bloco M, Sala 64-A, Uvaranas, Ponta Grossa, Paraná 84030-900 Brazil

**Keywords:** Orthodontics, Conversion degree, Dental Bonding, Light-Cured

## Abstract

**Objective:**

To evaluate the efficacy of a new orthodontic primer (Ambar APS Ortho; FGM Dental Products, Joinville, SC, Brazil) on shear bond strength (SBS) and degree conversion (DC) of metallic brackets bonding.

**Materials and Methods:**

240 sound maxillary premolars were randomized into 24 experimental groups based on: (1) Orthodontic primer (Ambar APS Ortho, Orthoprimer and Transbond XT); (2) Light-curing time (3-seconds and 10-seconds); (3) Light-curing unit (Valo Cordless and Quazar); and (4) Storage condition (immediate time [IT] and after thermocycling [TC]). After each storage time, specimens were subjected to SBS testing at a crosshead speed of 1 mm/min until failure, and values were recorded in MPa. For DC (%) analysis, adhesive discs were prepared and evaluated using micro-Raman spectroscopy at IT only. SBS and DC data were analyzed using four and four-way ANOVA, respectively and Tukey’s post hoc test (α = 0.05).

**Results:**

Ambar APS Ortho showed significantly higher SBS values across both light-curing units compared to all other groups (*p* = 0.0001). For DC, Orthoprimer showed the lowest values, while Ambar APS Ortho achieved the highest. Across all primers, a 10 s light-curing exposure resulted in significantly higher DC values compared to 3 s (*p* < 0.0001).

**Conclusions:**

Ambar APS Ortho exhibited superior performance in both SBS and DC, for both 3 s and 10 s light-curing times as well as IT or AT, supporting its reliability for bonding metallic orthodontic brackets.

**Graphical Abstract:**

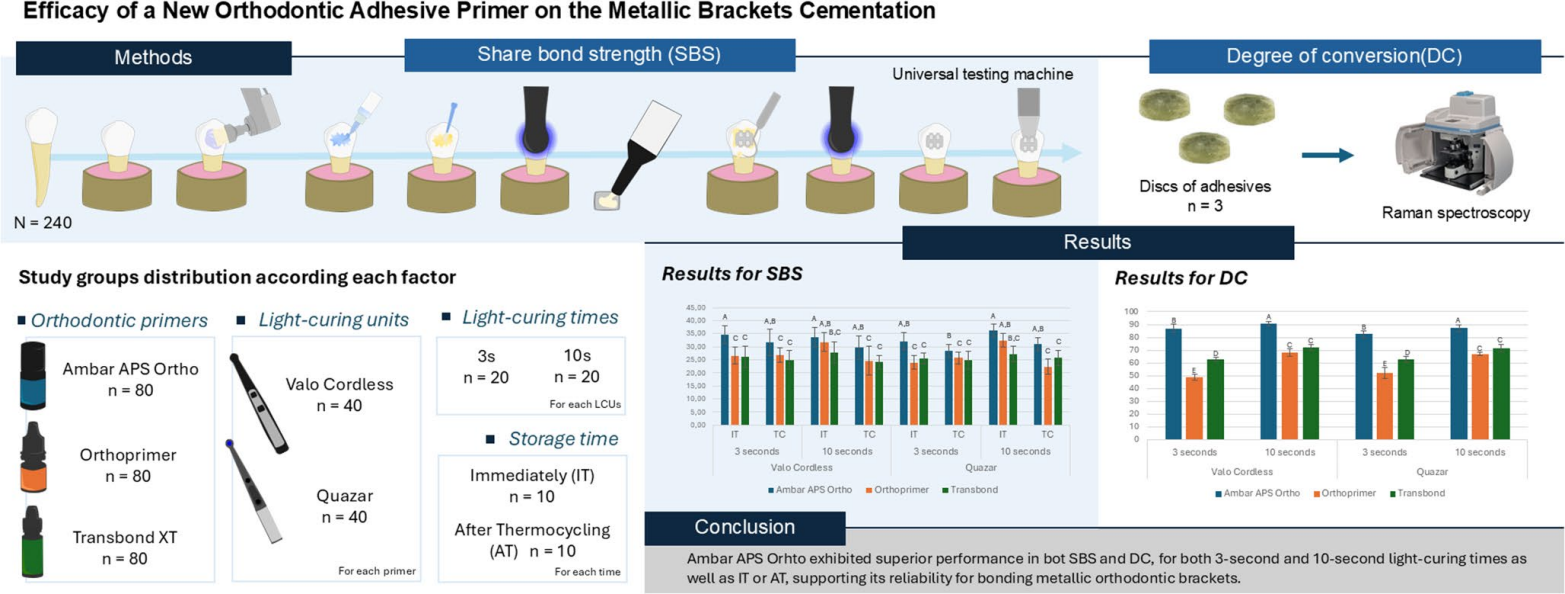

## Introduction

The tooth movement during orthodontic treatment critically depends on the forces exerted on wires and springs anchored to brackets [1]. Achieving effective and stable anchorage for this movement requires strong adhesion between the brackets and the tooth surface capable of withstanding both orthodontic and masticatory forces [2]. Unfortunately, literature reports a high incidence of bracket bonding failures, often associated with inappropriate material selection, handling errors, or improper application techniques [2, 3]. These failures can not only prolong treatment and increase clinical costs but may also cause irreversible damage to the enamel structure [4].

In the effort to optimize bonding protocols, modern light-emitting diodes (LEDs) curing units with polywave technology have introduced an “Ortho mode”. Manufacturers claim that this mode can achieve irradiance levels around 3200 mW/cm^2^, allowing for significantly shorter polymerization times during bracket bonding without compromising bond strength [5]. This time-saving feature is intended to reduce operator fatigue and enhance clinical efficiency, improving the overall experience for both clinicians and patients [6].

However, evidence on this approach remains controversial. Some authors [7–9] have shown that 3-second exposures may lead to lower shear bond strength (SBS) values when compared to longer exposures, such as 6 s. These differences have been partially attributed to a lower degree of conversion (DC) of the orthodontic adhesives when polymerized for shorter exposure durations [10, 11]. Nevertheless, exposure time is only one of several factors influencing the DC of light-cured materials [12, 13]. Other influential variables include the irradiance and radiance exposure of the light-curing units (LCUs), as well as the intrinsic reactivity and absorption characteristics of the photoinitiators used [13–16].

In this context, a promising approach to enhancing polymerization efficiency involves using alternative photoinitiators to the traditional camphoroquinone-amine system. Previous studies [13, 17, 18] have demonstrated that these alternative systems can be more reactive, leading to improved mechanical and physical properties of adhesive systems. Despite this potential, there has been limited interest from manufacturers in improving materials specifically developed for orthodontic bonding.

Recently, a novel orthodontic primer called Ambar APS Ortho (FGM Dental Group, Joinville, SC, Brazil) was introduced. This product features an Advanced Polymerization System (APS) that claims to achieve a high DC even with light exposure as short as 3 s at an irradiance of approximately 3000 mW/cm^2^ [19]. However, to the best of the authors’ knowledge, this new material has not yet been evaluated in comparison with commercially available orthodontic primers.

Another challenge is that high-power LCUs tend to be more expensive, as achieving high irradiance levels requires advanced internal technology and broader light guide tips to cover larger bonding areas [20]. These costs may ultimately impact on the affordability of clinical procedures. In response, a new low-cost, high-power LCU has been released (Quazar, FGM Dental Group, Joinville, SC, Brazil), claiming to offer the same irradiance as more expensive devices, thus raising the question of whether similar clinical outcomes can be achieved at lower costs. To the best of the authors’ knowledge, this novel LCU has not yet been evaluated against commercially available LCUs.

Thus, this in vitro study aims to evaluate the effectiveness of this new orthodontic primer in bonding metallic brackets when compared to other orthodontic primers, using 3 and 10 s curing times with two different LCUs. The null hypotheses tested were that the SBS and DC would not be affected by (1) the light-curing time, (2) the orthodontic primer used, (3) storage time (immediate vs. thermocycle), which applied only to SBS, and (4) the LCUs employed.

## Materials and methods

### Selection and teeth preparation

Two hundred and forty non-carious human maxillary premolars were used in this study. Teeth were collected following the acquisition of informed consent from the patients, in accordance with a protocol approved by the ethics committee of the State University of Ponta Grossa (protocol #7.560.064). After extraction, the teeth were disinfected in 0.5% chloramine, stored in distilled water at 4 °C, and used within six months to preserve sample integrity and reliability.

### Sample size calculation

Since the primary outcome of this study was the shear bond strength (SBS) of brackets bonded to maxillary premolars, the sample size calculation was based on the mean and standard deviation (17.3 ± 2.1 MPa) reported in a previous study [21] that used the same control group as the one proposed herein. With a significance level set at 5% and a statistical power of 80%, the initial calculation indicated the need for eight specimens per experimental group (G*Power software version 3.1, Heinrich Heine University Düsseldorf, Düsseldorf, NW, Germany). To compensate for potential specimen loss due to fractures, two additional teeth were included per group, resulting in a final sample size of ten premolars per experimental group (*n* = 10).

### Experimental design

**Fig. 1:**
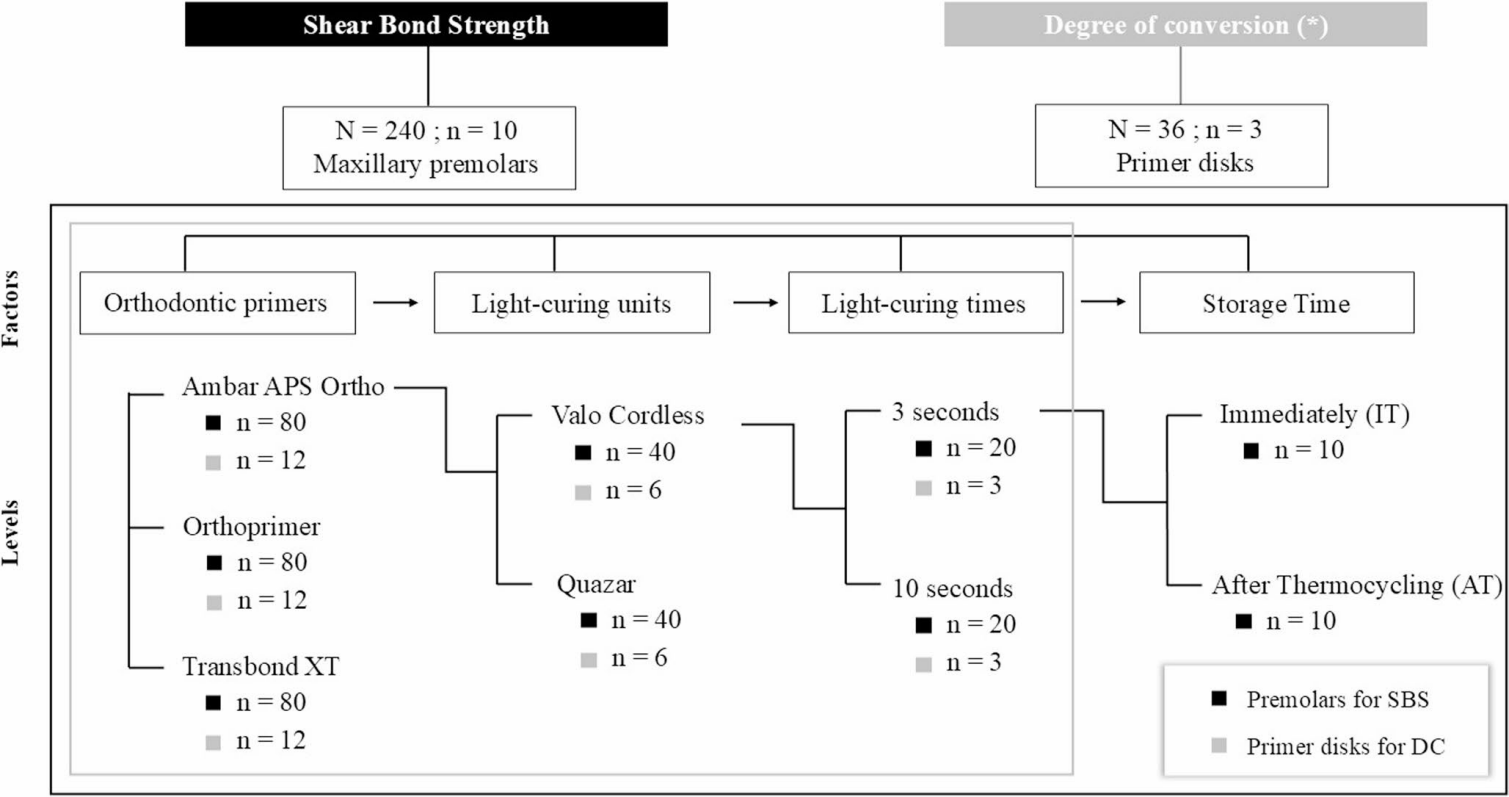
Experimental design. Distribution of samples for each experimental condition according to the factors analyzed in each test. Black squares represent groups for the shear bond strength test, while gray squares indicate groups for the degree of conversion test. (*) For the degree of conversion test, only orthodontic primers, light-curing units, and light-curing times were considered

Three orthodontic primers were evaluated: (1) Ambar APS Ortho, (FGM Dental Group, Joinville, SC, Brazil); (2) Orthoprimer (Morelli, Sorocaba, SP, Brazil); and (3) Transbond XT (Solventum, St Paul, MN, USA), which was used as the gold standard for orthodontic bonding. Each primer was tested using two different light-curing times (3 and 10 s) and two different LUCs [Valo Cordless (Ultradent Products Inc, South Jordan, UT, USA) and Quazar (FGM Dental Group, Joinville, SC, Brazil)]. All experimental conditions were evaluated both immediately (IT) and after thermocycling (AT). A total of 240 premolars were randomly allocated into 24 groups (*n* = 10 per group) based on the combinations of orthodontic primers, light-curing time, LCUs, and storage time. The experimental design is illustrated in Fig. 1.

### Bonding procedures

All the bonding procedure was performed by a single and calibrated operator, a specialist in Orthodontics. Each tooth was individually embedded in chemically cured acrylic resin (Vipiflash, São Paulo, SP, Brazil) within a 10 mm-high polyvinyl chloride tube, exposing only the crown. Details of the materials used, and their compositions are provided in Table 1.

**Table 1:** Materials and compositions

Materials	Manufacturer	Composition
Condac 37 - Phosphoric acid	FGM Dental Group, Joinville, SC, Brazil)	37% phosphoric acid
Ambar APS Ortho – Light Cure Orthodontic primer	FGM Dental Group, Joinville, SC, Brazil	10-MDP, methacrylate monomers, photoinitiators complex (APS), co initiators, stabilizers, Silica particles filler and ethanol.
Orthoprimer – Light Cure Orthodontic primer	Morelli, Sorocaba, SP, Brazil	Bis-GMA, TEGMA, HEMA, N, N-Dimethyl-p-toluidine, camphorquinone, 2;6 DI-ter-nutyl-p-cresol, 2-(Dimethylamino)ethyl methacrylate
Transbond XT - Light Cure Orthodontic primer	Solventum, St Paul, MN, USA	Bis-GMA, TEGDMA, 4-(Dimethylamino)-Benzeneethanol
Orthocem - Light Cure Orthodontic Resin	FGM Dental Group, Joinville, SC, Brazil	Methacrylate monomers (Bis-GMA, TEGDMA), methacrylate phosphatized monomers, stabilizers, camphorquinone, co-initiators, silicon dioxide filler and pigment.
Orthobond - Light Cure Orthodontic Resin	Morelli, Sorocaba, SP, Brazil	Bis-GMA, TEGDMA, HEMA-2, camphorquinone, Butylated hydroxytoluene, Dimethyl Propionic acid, Dimethyl aminobenzoic acid ethyl, Diphenyliodoniun Hexafluorophosphate, Dimethyl aminoethyl Methacrylate, Silicon dioxide, Aluminum dioxide.
Transbond XT - Light Cure Orthodontic Resin	Solventum, St Paul, MN, USA	Silane treated Quartz, Bis-GMA, Bisphenol A Dimethacrylate, Silane treated Silica, camphorquinone, Diphenyliodonium Hexafluorophosphate, Triphenylantimony.

(*) *10-MDP* 10-metehacryloyloxydecyl dihydrogen phosphate), *Bis-GMA* bisphenol-A glycidyl methacrylate, *TEGDMA*  triethyleneglycol dimethacrylate, *HEMA-2* 2-hydroxyethyl methacrylate

Initially, the specimens underwent prophylaxis with pumice paste and water for 30 s. This was followed by phosphoric acid etching (37% phosphoric acid, FGM Dental Products, Joinville, SC, Brazil) applied for 30 s, rinsing with water for 30 s, and air-drying for 10 s.

Bonding procedures were performed using a standardized technique. A thin layer of each primer was applied using a microbrush (Cavibrush, Regular size, FGM Dental Group, Joinville, SC, Brazil), followed by a gentle air-drying for 10 s. Light curing was then performed according to the group allocation: 3 s at ≈ 3.200 mW/cm^2^ or 10 s at ≈ 1000 mW/cm^2^. An orthodontic resin from the same manufacturer as the primer was applied to the metal brackets, which were then placed on the buccal tooth surface. Stainless steel maxillary premolar brackets with a Roth prescription (0° angulation, -7° torque, and a base area of 6.5 mm^2^) were used for all samples (Monobloc Roth; Morelli, Sorocaba, SP, Brazil). The orthodontic resin was dispensed into each bracket, which was then positioned on the buccal tooth surface and manually pressed into place. Excess resin was carefully removed using a dental probe. Final light-curing was performed for 40 s (10 s per bracket margin) using the LCU at 1000 mW/cm^2^, depending on the experimental group.

### Shear Bond Strength (SBS)

Half of the specimens treated were stored for 24 h in distilled water before the SBS test was performed. The other half specimens were submitted to 10,000 thermal cycles at 5 and 55 °C, with a dwell time of 1 min (OMC 350 TC Thermal Cycler, Odeme Biotechnology, Joaçaba, SC, Brazil). After that, the specimens were mounted on a universal testing machine adapted to shear testing (Instron, Canton, MA, USA) with a 1,000 N load cell. Each specimen was positioned at the bracket-enamel interface. The setup ensured alignment at the resin-enamel interface, guaranteeing correct shear forces between application between the chisel and the center of the load cell. The crosshead speed in the compressive mode was set at 1 mm/min until failure.

To determine SBS values, the failure load was divided by the bonding area. After bracket removal, the samples were analyzed by a blinded operator using a stereomicroscope (SZH-131, Olympus, Tokyo, Japan) to assess the Adhesive Remnant Index (ARI) on both the tooth surface and the bracket base. Subsequently, a subset of samples from each experimental group was randomly selected for field emission scanning electron microscopy (FE-SEM) analysis by the same researcher. For FE-SEM preparation, the buccal surface exhibiting residual resin was separated from the tooth crown to reduce specimen size. These fragments were mounted on aluminum stubs and stored in desiccators containing fresh silica gel for 24 h at 37 °C. Finally, the mounted specimens were sputter-coated with gold (MED 010, Balzers Union, LI-01, Liechtenstein) prior to examination with FE-SEM *(*MIRA 3 LM, Tescan Orsay Holding, Warrendale, PA, USA).

The ARI score was used to classify the failure modes according to the following criteria: Score 0 = no resin left on the tooth surface; Score 1 = less than half of resin left on the tooth surface; Score 2 = more than half of resin left on the tooth surface; Score 3 = all resin left on the tooth with distinct impression of the bracket base [22].

### Degree of Conversion (DC)

For this purpose, the following variables were evaluated: three primers were light-cured using two LCUs and subjected to different light-curing times. Three discs from each group were produced using a circular metal matrix (Ø 5.8 mm and 1.0 mm thickness) placed between two plastic strips. After applying a thin layer of petroleum jelly to isolate all parts of the matrix, a drop of primer was dispensed into the matrix cavity until it was filled, taking care to avoid bubble formation. An air stream was applied to the surface at 10 cm to facilitate solvent evaporation.

After covering the upper surface with a plastic strip, the primers were light-cured for either 3 s (≈ 3.200 mW/cm^2^) or 10 s (1.000 mW/cm^2^) using the different LCUs. The LCU tip was positioned as close as possible to the specimen surface. After polymerization, the discs were removed from the matrix and slightly polished with 600-grit SiC paper to eliminate the oxygen inhibited layer.

DC measurements were performed in a micro-Raman spectrometer (XploRA ONE, Horiba scientific, Piscataway, NJ, USA) which was first calibrated to zero and then using a silicon standard. The specimens were evaluated under the following parameters: neon laser with 632 nm wavelength at 20 mW power, spatial resolution of ≈ 3 μm, spectral resolution of ≈ 5 cm^− 1^, with accumulation time of 30 s and 3 co-additions, using 100x magnification (Olympus UK, London, UK) for a laser beam diameter of approximately 1 μm.

For DC comparison, spectra from unpolymerized primers were used as reference (data not shown). The acquired spectra were processed using Spectragryph software (version1.2.16.1; Spectroscopy Ninja, Berchtesgaden, BY, Germany) to determine the integrated area of the relevant peaks. The DC was calculated using the following formula: DC (%) = (1 - [R cured / R uncured]) × 100, where R represents the ratio between the aliphatic and aromatic **C = C** peak areas at 1639 cm^− 1^ and 1609 cm^− 1^, in cured and uncured primers [23, 24].

### Statistical analysis

The data were initially analyzed using the Kolmogorov-Smirnov test to assess normality of distribution, and Bartlett’s test was employed to evaluate the homogeneity of variances. After confirming data normality and variance homogeneity, the SBS data (MPa) were subjected to four-way ANOVA (light-curing time × light-curing unit × primer type × storage time). The ARI scores were analyzed using chi-square or Fisher’s exact test. Similarly, the DC data (%) were analyzed using a three-way ANOVA (light-curing time × light-curing unit × primer type). Tukey’s post hoc test was used for pairwise comparisons, with the significance level set at α = 0.05. All statistical analyses were performed using Statistica for Windows software (StatSoft, Tulsa, OK, USA). The FE-SEM images were evaluated qualitatively only.

## Results

### Shear Bond Strength (SBS)

Although the four-way ANOVA did not reveal a statistically significant main effect involving all four factors simultaneously (light-curing time × light-curing unit × orthodontic primer × storage time), a significant triple interaction was identified among primer type, light-curing time, and storage time (*p* < 0.0001; Table 2). Notably, the main factor ‘light-curing unit’, did not significantly influence the SBS values (*p* > 0.05), indicating no significant differences between Valo Cordless and Quazar (*p* > 0.05; Table 2).

**Table 2:** Means and standard deviations of shear bond strength (MPa) obtained for each experimental condition, as well as statistical analysis (*)

	Valo Cordless				Quazar			
Orthodontic Primers	3 seconds		10 seconds		3 seconds		10 seconds	
	IT	TC	IT	TC	IT	TC	IT	TC
Ambar APS Ortho	34.7 ± 3.4 A	31.7 ± 4.9 A,B	33.7 ± 3.7 A	29.8 ± 4.5 A,B	32.1 ± 3.3 A,B	28.4 ± 2.4 B	36.3 ± 2.4 A	31.0 ± 2.5 A,B
Orthoprimer	26.6 ± 3.3 C	26.9 ± 2.8 C	31.8 ± 3.6 A,B	24.7 ± 5.4 C	24.0 ± 2.7 C	25.7 ± 2.2 C	32.5 ± 2.6 A,B	22.3 ± 2.9 C
Transbond XT	26.2 ± 4.0 C	24.9 ± 3.6 C	27.8 ± 4.2 B,C	24.1 ± 2.6 C	25.5 ± 2.2 C	24.8 ± 3.5 C	24.8 ± 3.5 C	25.7 ± 2.8 C

(*) Different uppercase letters indicate statistically significant differences among experimental groups (Tukey’s test, p < 0.05)

IT = immediate testing; TC = after thermocycling (10,000 cycles, 5/55 °C)

Regarding light-curing time, the Orthoprimer group light-cured for 10 s showed significantly higher SBS values under IT condition compared to the 3 s exposure, regardless of the LCU used (*p* < 0.0001; Table 2). However, after TC, no significant differences in SBS were found between the two light-curing times (*p* > 0.05; Table 2).

With respect to the primer type, Ambar APS Ortho consistently demonstrated the highest SBS values among the tested primers (Table 2; *p* = 0.001), except when compared to Orthoprimer under the IT condition with a 10 s light-curing exposure time for both LCUs (Table 2; *p* > 0.05). In general, the other primers (Orthoprimer and Transbond XT) showed similar SBS results in most experimental conditions (Table 2; *p* > 0.05).

When comparing storage conditions (IT vs. TC) within each light-curing time, significant reduction in SBS after thermocycling were observed only in specimens light-cured for 10 s. Specifically, Orthoprimer showed a significant reduction in SBS following thermocycling after 10 s of light exposure (*p* < 0.00001; Table 2).

The ARI data presented in percentage (%) for each experimental condition, are shown in Table 3, and representative FE-SEM images of the ARI patterns observed in this study are presented in Fig. 1. No significant differences were found regarding the ‘light-curing time’,‘light-curing unit’, or ‘storage condition’ (*p* > 0.06; Table 3). Only a significant difference was observed for the ‘orthodontic primer. Specifically, Ambar APS Ortho showed a significantly higher frequency of ARI scores from 1 to 3 compared to Orthoprimer and Transbond XT (p < 0.01; Table 3), indicating a greater amount of resin remaining on the tooth surface after bracket debonding.

**Table 3:** Percentage (%) of Adhesive Remnant Index (ARI) scores for each experimental condition using both light-curing units

	Valo Cordless																Quazar															
OrthodonticPrimers	3 seconds								10 seconds								3 seconds								10 seconds							
	IT				TC				IT				TC				IT				TC				IT				TC			
	0	1	2	3	0	1	2	3	0	1	2	3	0	1	2	3	0	1	2	3	0	1	2	3	0	1	2	3	0	1	2	3
Ambar APS Ortho	50	50	0	0	70	10	10	10	40	50	10	0	70	30	0	0	80	20	0	0	60	40	0	0	20	30	10	40	50	30	20	0
Orthoprimer	60	40	0	0	80	10	10	0	80	20	0	0	90	10	0	0	90	10	0	0	90	10	0	0	90	0	0	10	70	10	20	0
Transbond XT	40	20	4 0	0	70	30	0	0	30	30	0	40	60	40	0	0	90	10	0	0	80	20	0	0	100	0	0	0	100	0	0	0

### Degree of Conversion (DC)

Three-way ANOVA statistical analysis revealed no significant differences of triple interaction (light-curing time × light-curing unit × orthodontic primer) or the double interactions (light-curing unit × orthodontic primer and light-curing time × light-curing unit), as well as the main factor ‘light-curing unit’ (*p* > 0.12; Table 4). However, the double interaction ‘light-curing time × orthodontic primer’ exhibited significant differences on DC (*p* < 0.0001; Table 4). It is important to note that the ‘light-curing unit’, as an isolated factor, did not significantly affect the DC values (*p* > 0.05), demonstrating that Valo Cordless and Quazar performed similarly under the tested conditions (*p* > 0.05; Table 4).

**Table 4:** Means and standard deviations of degree of conversion (%) obtained in each experimental condition (*)

Orthodontic Primers	Valo Cordless		Quazar	
	3 s	10 s	3 s	10 s
Ambar APS Ortho	86.9 ± 3.5 B	90.8 ± 1.8 A	82.5 ± 2.6 B	87.1 ± 2.7 A
Orthoprimer	48.6 ± 2.3 E	68.2 ± 2.9 C	52.1 ± 4.2 E	67.1 ± 1.4 C
Transbond XT	62.7 ± 1.6 D	72.2 ± 2.2 C	62.8 ± 2.4 D	71.3 ± 3.2 C

(*) Means identified with the same capital letter are statistically similar. (Tukey’s test, *p* ≥ 0.05)

Ambar APS Ortho exhibited the highest DC values across all experimental groups, whereas Orthoprimer consistently showed the lowest (*p* < 0.0001; Table 4). The other primers demonstrated intermediate DC values. Importantly, increasing the light-curing time to 10 s significantly improved the DC values for all primers when compared to the 3 s exposure (*p* < 0.0001; Table 4). Following the 10 s light-curing protocol, Ambar APS Ortho achieved statistically similar and higher DC values, while Orthoprimer and Transbond XT presented statistically similar but lower DC values (*p* < 0.0001; Table 4).

## Discussion

The exposure reciprocity law posits that delivering a similar radiant exposure (J/cm^2^) should yield equivalent material properties, regardless of the light-curing time [10, 25]. Consequently, no significant differences in SBS or DC were initially expected, given that both LCUs were programmed to deliver comparable radiant exposures according their manufacturers: (i) the high irradiance mode, delivering 9.6 J/cm^2^ (Valo Cordless; 3s at 3200 mW/cm^2^) and 9.0 J/cm^2^ (Quazar; 3s at 3000 mW/cm^2^), and (ii) the standard mode, delivering 10 J/cm^2^ for both LCUs (10s at 1000 mW/cm^2^) [19, 26]. However, the exposure reciprocity law was not upheld in this case. Although the Ambar APS Ortho and Transbond XT groups show no significant differences in their SBS values between light-curing times, all evaluated primers exhibited significantly higher DC when light-cured for 10 s. These findings lead to the rejection of our first null hypothesis.

One potential explanation for this deviation from the reciprocity law is the discrepancy between the manufacturer’s reported irradiance and the actual energy emitted by the LCUs [27, 28]. For the specific LCUs used herein, Guarnieri et al. [27] reported that the delivered energy was not only different between the LCUs (6.5 J/cm^2^ for Valo Cordless vs. 8.2 J/cm^2^ for Quazar at 3 s) but also increased disproportionately at 10 s to 9.2 J/cm^2^ and 10.7 J/cm^2^, respectively, further complicating a direct comparison based on reciprocity law [10].

While these physical variations in energy delivery contribute to these outcomes, they alone cannot account for the significant performance differences among groups. Therefore, it is crucial to consider intrinsic material factors known to influence polymerization kinetics, such as the photoinitiator system and filler content [29–31]. These compositional variables likely underline the performance difference observed not only between light-curing times but also among the different primers, thus leading to the rejection of our second null hypothesis.

Primers differ from conventional restorative adhesives due to their specific formulation, characterized by low viscosity, a high solvent concentration, and low molecular weight monomers with minimal filler content [32]. While these adaptations are designed to optimize the wetting and penetration on etched enamel [33, 34], they also create an adverse scenario for polymerization, particularly for adhesive systems content camphorquinone (CQ) as the primary photoinitiator [29, 30, 35]. The low viscosity, intensified by solvents, facilitates a high mobility of reactive species within the adhesive matrix [35]. When subjected to high irradiance light-curing, the resulting instantaneous and high concentration of free radicals in this mobile environment significantly increases the probability of biradical termination, an event where radicals mutually neutralize each other instead of propagating the polymer network [31].

This premature termination mechanism explains why the Transbond XT and Orthoprimer, based in CQ and low molecular weight monomers such as TEGDMA, demonstrated significantly lower DC values when light-cured for 3 s. Notably, the Orthoprimer, which also contains the highly hydrophilic monomer HEMA, exhibited the lowest DC at 3 s when compared with the other groups and required a 10 s exposure to significantly improve both its DC and SBS. Consequently, CQ-based and highly hydrophilic adhesive systems, which are particularly susceptible to biradical termination, require longer exposure times. This allows for a more gradual activation of radicals, ensuring that propagation kinetics can overcome the high rate of termination, resulting in a more robust, cross-linked polymer network and achieving adequate monomer conversion [31, 35].

However, although it is generally expected that improvements in DC would be reflected in enhanced bonding properties, this direct correlation appears to exist primarily in in situ models, where the complex interactions among the various components of the adhesive interface could influence these variables [23].

Indeed, the Transbond XT group, despite showing improvements in DC between the different light-curing times and a generally higher DC than Orthoprimer, did not present differences in SBS under any condition. It should be noted that Transbond XT is considered the gold standard for bracket cementation, owing to its well-documented high performance under different scenarios [6–9, 36]. Nevertheless, our findings, in line with studies such as that of Pithon et al. [37] demonstrate that primers like Orthocem can exhibit comparable performance. In fact, when considering the ideal individual SBS value required to withstand masticatory forces (> 25.8 MPa) [9], both primers demonstrated satisfactory performance under the conditions evaluated herein.

However, the Ambar APS Ortho was superior under all evaluated conditions and showed no significant performance differences between the light-curing times. Due to the exact formulation is proprietary, previous studies [17, 18] suggest that the enhanced properties of APS-containing adhesives are due to kinetic behavior analogous to other alternative photoinitiators. These systems are proposed to feature a CQ-recycling mechanism, where a single molecule generates multiple radicals. This process efficiently overcomes the biradical termination phenomenon that typically limits low-viscosity systems [38].

Whereas conventional CQ-based systems reach their maximum reaction rate at very early stages of conversion (< 15%) and thus vitrify prematurely, advanced photoinitiators like those in the APS delay this point until a much higher degree of conversion is achieved (> 50%). This delayed vitrification allows a significant portion of the polymerization shrinkage to occur while the adhesive agent is still in a more fluid, gel-like phase, thereby dissipating stress before it can compromise the bond [35]. Thus, the APS not only achieves a higher degree of polymerization but does so through more favorable mechanics. This system is particularly effective under high irradiance, as it can efficiently utilize the high photon flux to accelerate the reaction and reach a high final DC in a short time, without being negatively impacted by biradical termination [38].

Although the Ambar APS Ortho group exhibited a higher DC when cured for 10 s, its 3 s values were superior to the other primers, and no significant differences in SBS were observed between the two light-curing times. This equivalence in SBS can be explained by two factors. First, it is likely that the DC achieved by Ambar APS Ortho in just 3 s had already surpassed the necessary conversion threshold to yield the high SBS values observed. Thus, the additional increase in DC at 10 s, while statistically significant, may represent a point of diminishing returns in terms of its impact on SBS [23, 30, 35]. Second, the quality of the resulting polymer network may be more influential than the absolute final DC value [35]. As previously discussed, the favorable kinetics of the APS would result in a network with lower internal stress, regardless of the light-curing time. Both hypotheses could, from another perspective, justify the lack of a direct DC-SBS correlation for this primer.

Although the intrinsic characteristics of orthodontic primers, as previously discussed, seem contrary to the superior results obtained with Ambar APS Ortho, their very good performance can be explained by a synergy between its monomeric formulation and the APS system [17, 35, 38]. On one hand, the formulation must counteract the low viscosity induced by diluent monomers such as TEGDMA through an optimized balance with more viscous and hydrophobic monomers like Bis-GMA, which improve polymerization kinetics by restricting radical mobility [31, 35]. Concurrently, the presence of 10-MDP monomer provides a highlight advantage by leveraging the effects of the pre-etched surface. Acid etching increases the negative charge of enamel surface, which increases the availability calcium ions on the enamel, allowing 10-MDP to form a more robust and stable chemical bond at the adhesive interface [39, 40].

Similarly, the Ambar APS Ortho group maintained the highest SBS values after thermocycling (TC) without showing significant degradation. These results are consistent with the high long-term stability observed for the APS in the study by Moreira et al. [17]. In contrast, the Orthoprimer group was the only one to show a significant decrease in SBS values when light-cured for 10 s. That is, even though it was the only group to improve with the increased light-curing time, these higher values were not maintained after aging, demonstrating a susceptibility to thermal and hydrolytic stress. Thus, both the Transbond XT and Ambar APS Ortho groups, which presented better DC values, showed no significant differences after aging. The stability of these materials against TC suggests greater resistance to hydrolytic degradation over time. These findings, which demonstrate that long-term stability is material-dependent, lead to the rejection of our third null hypothesis.

In this study, no significant differences were observed in either the SBS or DC results when the two LCUs (Valo Cordless and Quazar) were used; therefore, the fourth null hypothesis was accepted. These findings could be contradictory with differences between energy delivery as previously explained [27]. The lack of differences in outcomes suggests that both radiant exposure values were sufficient to polymerize these orthodontic primers at point where the SBS and DC values were not shown differences. This implies that while differences in LCU output exist, they may not always translate to significant differences in performance, especially when using highly reactive adhesive systems. Nevertheless, it reinforces that lower-cost LCUs can offer outcomes equivalent to more expensive devices, provided their actual radiant exposure is verified as sufficient for the clinical task [20].

The high SBS values observed in this study, exceeding 25 MPa, should be interpreted in light of current trends in minimally invasive orthodontics [41–43]. While such values may provide sufficient strength to withstand peak masticatory forces [9], preservation of enamel integrity during debonding remains a primary clinical concern.

**Fig. 2:**
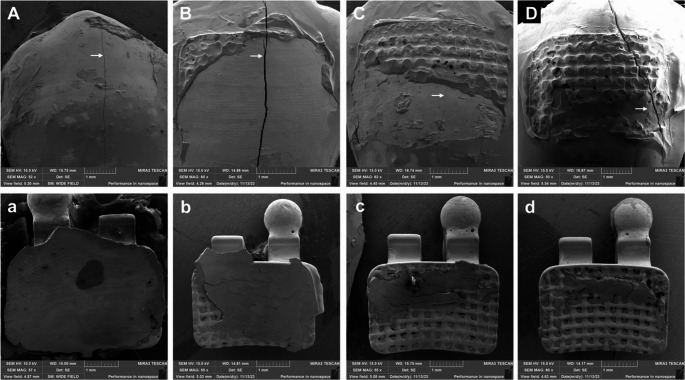
Representative FE-SEM images of each Adhesive Remnant Index (ARI) scores. Capital letters correspond to fracture patterns of adhesive remnants on the enamel surface following SBS test, whereas lowercase letters illustrate remnants on the bracket base from the same sample. The specific ARI scores are as follows: (**A**, **a**) = 0, (**B**, **b**) = 1, (**C**, **c**) = 2, and (**D**, **d**) = 3. The fracture lines visible in images A, B and D (white arrow) are attributed to artifacts induced during the sputter coating procedure due to vacuum effects on desiccated specimens

In the present study, the predominant failure mode corresponded to an ARI score of 0 (Table 3), indicating failures at the enamel–adhesive interface. However, neither failure mode analysis nor FE-SEM observations revealed evident enamel damage (Fig. 2). Although some studies suggest that ARI scores of 0 may be associated with an increased risk of enamel damage [36, 44], their clinical relevance remains controversial [45]. To date, there is no conclusive clinical evidence directly linking specific ARI patterns to adverse enamel outcomes. Nevertheless, it should be recognized that orthodontic debonding procedures may induce subtle microscopic alterations in enamel structure, regardless of the adhesive system used [46]. Therefore, achieving an optimal balance between adequate bond strength and enamel preservation remains a key objective in contemporary orthodontic research.

Although this study provides valuable information on the performance of this new orthodontic primer, the limitations inherent to in vitro models should be considered. These include the restricted selection of orthodontic primers for comparison, the exclusive use of sound enamel as the bonding substrate, and the absence of additional aging protocols, such as mechanical fatigue or acid challenge. Furthermore, as a detailed quantification of enamel surface topography was beyond the scope of this research, future investigations should focus on the balance between adhesive performance and preservation of dental tissues. Finally, the complexity of the oral environment, including variations in pH, masticatory forces, and microbiota, may influence material performance in ways that cannot be fully replicated in the laboratory. Therefore, well-designed clinical trials are required to validate these findings under the biological and functional conditions of the oral environment, ensuring not only reliable bond strength but also evaluating their potential impact on enamel surface integrity.

## Conclusion

Within the limitations of this study, it is possible to conclude that the Ambar APS Ortho primer exhibits superior bonding properties compared to the other primers tested herein, even when polymerized for 3 s, maintaining high SBS values, including after thermocycling, also, no differences were showed between Valo Cordless and Quazar light-curing units. 

## Data Availability

The data that support the findings of this study are available from the corresponding author upon reasonable request.
